# Cause, Nature, and Treatment of Western Fevers

**Published:** 1846-06

**Authors:** 


### SELECTIONS

FROM

### AMERICAN AND FOREIGN JOURNALS.

---

*Cause, Nature, and Treatment of Western Fevers.*—Dr. Hunt, of Wisconsin, has the following remarks on this subject in the Boston Medical and Surgical Journal. Everything written concerning Western fevers has interest for those who practice physic where they prevail. Among the original communications in this number we publish the speculations of Dr. Harris, touching malaria, and the *modus operandi* of febrifuges. Dr. Hunt's views on the same subjects are given in a summary style; it will be interesting to compare the conclusions of two writers who have studied the phenomena of the same disease a thousand miles apart.—Eds.

1. Marsh miasm is a subtle virus, and the material agent that produces the various (so called) bilious fevers of the West.

2. This poison is inhaled, enters the circulation, and acts as a disturbing cause in the system of nutrition.

3. It destroys the healthy balance between composition and decomposition, or assimilation and metamorphosis, diminishing or entirely suspending the former, and morbidly increasing the latter.

4. The metamorphosis of the vital tissues does not result in the formation of cholic acid and urate of ammonia, or proper bile and urine, but in an *abnormal* transformation.

5. The transformation of the living organism does not consist in the oxidation of its constituent elements, and the consequent formation of carbonic acid matter and animal heat, as is the case in inflammatory fevers and those of high reaction.

6. For want of centrifugal force, or in consequence of the sedative influence of the poison, this mass of transformed animal matter with the blood is accumulated in the central vascular system, and in consequence of a deficient hæmatosis the blood is rendered dark and thick.

7. Congestion consists in an overloaded state of the vascular system with this impure blood, and an engorged and obstructed condition of the liver, and a consequent determination of blood to the head.

8. The brain, in all cases of portal congestion and hepatic obstruction, suffers in consequence of a deficient inferior circulation, while the balance is thrown upon the brain, constituting what might with propriety be called *hyperæmia of the encephalon*, which may, and in fact often does terminate in inflammation of this organ in fatal cases of congestive fever.

9. There are three grand indications to be fulfilled in the treatment of congestive fever, whether of the intermittent, remittent, or continued form; viz: 1, recall the circulation to the surface and extremities; 2, stop the *morbid* metamorphoses of the vital tissues; 3, unload the liver and keep up a free discharge of "bilious matter"(?) until the blood is renovated.

10. The first indication is fulfilled by the use of stimulants, externally and internally.

11. The second, by the introduction into the system of some eutrophic agent, such as quinine, salacine, arsenic, &c., which will act on the system of nutrition, neutralizing the poison or fortifying the vital powers against the chemical, or the force of assimilation against that of transformation, and thereby restore the balance between these two opposing movements in the animal economy.

12. The third and last indication is fulfilled by emetics, deobstruents and laxatives. Ipecac. makes the best emetic; calomel the best deobstruent; castor oil, rhubarb, and jalap, the best cathartics.

13. Drastic cathartics should generally be avoided, especially in the first stage of the disease. They do hurt by irritating the mucous membrane of the alimentary canal, and therefore increasing the congestion by diminishing the centrifugal force, or rather by increasing the centripetal; also by producing a serous discharge from the portal capillaries without acting particularly upon the liver, thereby rendering the blood in the portal vessels more thick and gluey, which is already too much so to circulate readily through this organ. At the same time severe purging tends powerfully to debilitate the patient, without the least possible gain in the first stage, except it unloads the bowels of fecal matter. But in the latter stage of the disease, after the "bilious matter" is discharged in large quantities into the bowels, it is necessary to give pretty active and frequent cathartics.

14. Profuse perspiration is of no use, but on the contrary it thickens the blood and debilitates the patient.

*Note.*—I have known patients, almost destitute of fat, laboring under congestive fever, to lose five pounds in weight a-day for several days in succession; and the only secretions that were materially increased, were those of the liver and bowels. The discharge was homogeneous, mostly black, and of a semi-fluid consistency. The respiration was slow and oppressed, and the surface and extremities cool; the pulse slow and soft. Now if the tissues did not pass off through the liver and bowels, where did they find their exit?

